# An Interesting Family

**Published:** 1876-09

**Authors:** John E. Walker


An Interesting Family.—We are in receipt of the following interesting letter from Dr. John E. Walker, a prominent physician of Greenesboro, Ga. In addition to the cases mentioned by the Doctor, we would in this connection call attention to the case published some years ago in this journal, where the husband returned from the army with his arm amputated. He was nursed by his wife, who was pregnant. The child was born with one arm amputated or absent on the same side as the father's amputated arm, at the same point, and presenting much the same appearance:

*Dr. W. F. Westmoreland,*
*Editor Medical & Surgical Journal:*

"I have just met with a colored woman—Nancy Park, æt. 62—who has been totally blind from birth. She is the sixth child of healthy parents, who had in all ten children. Nancy married young, and had her first child at twenty years old, and altogether fourteen children. The fifth child was born with one eye only, the sixth was totally blind, and the tenth had but one eye; the rest were all free from defect. Nancy has a large number of grand-children, all of whom have two good eyes each. Nancy's parents accounted for her blindness by her mother being badly frightened during her pregnancy, at seeing a girl who had been badly burned about the face and eyes, making her a frightful object to behold; so much so that the mother covered her face, lest her child be *marked.* All this may pass for what it is worth; the child was certainly born blind. The ball of one eye is shrunken, the humors all absent; the other is spherical, with contracted but prominent cornea; the humors condensed and opaque; the eyes have never given pain or other trouble.

"I have given you the facts, most of which have come under my personal observation. I have been acquainted with the family for many years. I have no comments to make, other than that I do verily believe that the mental impressions of the mother *sometimes* affect the fœtus in utero. My classmate, Dr. J. H. Oliver, of Burke county, Georgia, reported a case in the *Southern Medical and Surgical Journal*, about the year 1850 (my *Journal* is misplaced, and I write from memory) in which the proof seems irresistible. He writes that he treated a gentleman (whose wife was about half advanced in her pregnancy) for some affection of the head, for which he applied leech cups to the temples. When the infant was born, it had on each temple the impression of the rim of the cups, and the number of scars corresponding to the blades of the scarificator. I might cite other cases, but need not. If it was not the effect of the mother's mind in this case, how can it be accounted for? It could not be accidental. Well, then, if it holds good in the latter, may it not be true in the case of Nancy's mother?

JOHN E. WALKER.

